# A moment kernel machine for clinical data mining to inform medical decision making

**DOI:** 10.1038/s41598-023-36752-7

**Published:** 2023-06-28

**Authors:** Yao-Chi Yu, Wei Zhang, David O’Gara, Jr-Shin Li, Su-Hsin Chang

**Affiliations:** 1 Department of Electrical and Systems Engineering, Washington University in St. Louis, St. Louis, MO 63130 USA; 2 Division of Computational and Data Sciences, Washington University in St. Louis, St. Louis, MO 63130 USA; 3 Division of Biology and Biomedical Sciences, Washington University in St. Louis, St. Louis, MO 63130 USA; 4 Division of Public Health Sciences, Department of Surgery, Washington University School of Medicine, St. Louis, MO 63110 USA

**Keywords:** Medical research, Outcomes research

## Abstract

Machine learning-aided medical decision making presents three major challenges: achieving model parsimony, ensuring credible predictions, and providing real-time recommendations with high computational efficiency. In this paper, we formulate medical decision making as a classification problem and develop a moment kernel machine (MKM) to tackle these challenges. The main idea of our approach is to treat the clinical data of each patient as a probability distribution and leverage moment representations of these distributions to build the MKM, which transforms the high-dimensional clinical data to low-dimensional representations while retaining essential information. We then apply this machine to various pre-surgical clinical datasets to predict surgical outcomes and inform medical decision making, which requires significantly less computational power and time for classification while yielding favorable performance compared to existing methods. Moreover, we utilize synthetic datasets to demonstrate that the developed moment-based data mining framework is robust to noise and missing data, and achieves model parsimony giving an efficient way to generate satisfactory predictions to aid personalized medical decision making.

## Introduction

Surgery, as a major medical intervention, is usually considered when other treatments result in unsatisfactory outcomes. Predicting adverse events following surgery based on patients’ presurgical clinical data such as electronic health record (EHR) data is of crucial importance to inform both physicians and patients for decision making^1,2^. In recent years, the increased availability of clinical data and computing power greatly stimulated the development of machine learning (ML) techniques to extract information from clinical data. In particular, ML algorithms have made significant strides in AI-assisted medical procedures for preoperative prediction of postsurgical outcomes through EHRs^3,4^. The general ML problem focuses on finding an appropriate function *f* mapping each input data point ${\textbf{X}}$ to the desired output ${\textbf{y}}$, i.e.,

1 $$\begin{aligned} {\textbf{y}} = f({\textbf{X}}). \end{aligned}$$

This task is particularly challenging for datasets containing clinical records with a large size and mixed types of data, including diagnoses, treatments, vital signs, and laboratory values^5^.

In the past decade, numerous ML-aided methods have been proposed to assist medical decision making through the prediction of postsurgical events. For example, for weight-loss surgery, notable contributions include the application of logistic regression (LR) and Poisson regression (PR) to estimate the readmission rate^6^, the utilization of neural networks (NNs) and gradient-boosting machines (GBMs) to predict gastrointestinal leak and venous thromboembolism^7,8^, and the development of the super learner algorithm to predict the risk of 30-day readmission after bariatric surgery^9,10^. In addition to assessing possible postsurgical events, ML methods have been widely applied to identify abnormalities in medical images such as precancerous or premalignant lesions^11–14^. Primary examples range from a deep learning approach to mortality prediction for patients with coronary heart disease and heart failure^15^ to quantitative image feature extraction methods for the prognosis of early revascularization in patients with suspected coronary artery disease^16^. Algorithmically, deep neural networks have been attractive to medical researchers and practitioners, due to their ability to discover hidden structures in large datasets, leading to a high probability of achieving satisfactory results under suitable conditions^17^. Among these works, the integration of ML-techniques into medical research, although successful in many ways, usually suffers from low computational efficiency due to the heterogeneous structure, e.g., due to sparsity and irregularity, and the large size of clinical data^18^. In general, the complexity of ML algorithms grow exponentially in time and memory usage as a function of data size. Moreover, to produce better performance, deep neural networks further sacrifice robustness to noise and model parsimony, in addition to computational efficiency^19^.

Aiming to construct a parsimonious and computationally efficient model to assist medical decision making, particularly for surgical treatments, we develop a *moment kernel machine* for clinical data mining. The main idea is to introduce the notion of moments for clinical data to efficiently characterize patients’ overall health status. We further integrate the Hilbert Schmidt Independence Criterion (HSIC) Lasso method into the data preprocessing procedure. This leads to two major advantages: (1) the moment representation can quantitatively identify the crucial predictors impacting surgery outcomes; and (2) the dimension of the EHR data is significantly reduced, which facilitates its high computational efficiency in data analytic tasks. We then formulate medical decision making problems as ML classification problems, in which the moment representations extracted from EHR data are used as features for ML classifiers. To demonstrate, we choose three ML classifiers, LR, NNs, and GBMs, and used three clinical datasets to illustrate the generalizability of the developed moment kernel machine, that is, making medical decisions based on moments is valid for different clinical data regardless of the choices of the ML classifiers. We compare the classification performance resulting from our method with that from existing feature extraction methods, highlighting the model parsimony and high computational efficiency of the developed moment kernel machine. Furthermore, we demonstrate the robustness of moment kernel machine to noise and data loss using synthetic data.

## Methods

In this section, we first illustrate how clinical decision making can be assisted through machine learning algorithms to give informed predictions based on the patient’s clinical data. In particular, we formulate this task as a classification problem. Next, as the core of this section, we develop a novel moment kernel to extract features from the clinical data for the classification problem. Our method uniquely integrates the HSIC Lasso algorithm to select informative features, thereby improving computational efficiency without sacrificing classification performance. To demonstrate the applicability of the developed medical decision making machine, we also present case studies using both synthetic data and real clinical data.

### Event prediction as classification problems

Making decisions regarding a major medical intervention for a patient, e.g., deciding whether the patient should have surgery, generally requires (1) collecting sufficient data on possible post-intervention outcomes; (2) monitoring the current health condition and reviewing the medical history of the patient; and (3) assessing the significant factors influencing the possible outcomes based on the patient’s current health condition and medical history. This pipeline closely follows the formulation of a classification task in machine learning, where each class represents one possible post-intervention outcome. Then, the classification outcome obtained by the ML procedure using the patient’s clinical data can inform the medical decision.

In ML, features play a critical role in its performance. To ably assist with the medical decision making process, it is crucial to extract features from clinical data which reflect the patients’ medical conditions as well as ones that affect post-intervention outcomes, which is the main focus of the following sections.

### Moment kernel for clinical data

When working with datasets comprising both numerical and categorical values, we apply one-hot encoding^20^, a technique that converts categorical predictors to non-negative binary values. Each category is given a unique numerical representation as a feature vector consisting of entries of 0 and 1. We then pre-normalize each feature (predictor) within the training and testing datasets to ensure that all of them are in the interval [0, 1] before further normalizing the predictor vector of each patient to a probability distribution. Let ${\textbf{x}}_i=(x_{i1},\dots ,x_{iM})$ denote the predictor vector of the $i^{\textrm{th}}$ patient for $i=1,\dots ,N$, where *N* is the total number of patients and *M* is the number of predictors for each patient, then we normalize each ${\textbf{x}}_j$ as

2 $$\begin{aligned} p_{ij}=\frac{x_{ij}}{\sum _{j=1}^Mx_{ij}}, \end{aligned}$$

which yields a vector ${\textbf{p}}_i=(p_{i1},\dots ,p_{iM})$ satisfying $\sum _{j=1}^Mp_{ij}=1$. In addition, every component $p_{ij}$ of ${\textbf{p}}_i$ takes values in the same interval [0, 1], and this resolves any heterogeneity in the data, that is, different components of the predictor vector ${\textbf{x}}_i$ are drawn from different ranges. The property $\sum _{j=1}^Mp_{ij}=1$ then reveals that ${\textbf{p}}_i$ is a probability vector, and hence each ${\textbf{p}}_i$ represents the probability distribution of some random variable ${\textbf{A}}$. In particular, if ${\textbf{A}}$ takes values on the set $\Omega =\{\alpha _1,\dots ,\alpha _M\}$ containing *M* distinct elements, then the probability of the event $\{{\textbf{A}}=\alpha _j\}$ is given by ${\mathbb {P}}({\textbf{A}}=\alpha _j)=p_{ij}$ for each $j=1,\dots ,M$.

By the Hausdorff moment problem^21^, the probability distribution ${\textbf{p}}_i$ of ${\textbf{A}}$ is uniquely determined by the *moment vector* ${\textbf{m}}_i=(m_{i0},\dots ,m_{i,M-1})\in {\mathbb {R}}^M$, whose $k^{\textrm{th}}$-component is given by

3 $$\begin{aligned} m_{ik}={\mathbb {E}}({\textbf{A}}^k)=\sum _{j=1}^M\alpha _{j}^kp_{ij}, \end{aligned}$$

and referred to as the $k^{\textrm{th}}$-*moment* of the random variable ${\textbf{A}}$ with respect to the probability distribution ${\textbf{p}}_i$. Computationally, the moment vector ${\textbf{m}}_i$ can be easily obtained by ${\textbf{m}}_i={\textbf{p}}_i{\mathbb {M}}$, where

4 $$\begin{aligned} {\mathbb {M}}=\left[ \begin{array}{ccccc} 1 &{} \alpha _{1} &{} \alpha _{1}^2 &{} \cdots &{} \alpha _{1}^{M-1} \\ 1 &{} \alpha _{2} &{} \alpha _{2}^2 &{} \cdots &{} \alpha _{2}^{M-1} \\ 1 &{} \alpha _{3} &{} \alpha _{3}^2 &{} \cdots &{} \alpha _{3}^{M-1} \\ \vdots &{} \vdots &{} \vdots &{} \ddots &{} \vdots \\ 1 &{} \alpha _{M} &{} \alpha _{M}^2 &{} \cdots &{} \alpha _{M}^{M-1} \end{array}\right] \in {\mathbb {R}}^{M\times M} \end{aligned}$$

is the $M\times M$ Vandermonde matrix generated by the vector $\alpha =(\alpha _1,\dots ,\alpha _M)$ consisting of the possible values of the random variable ${\textbf{A}}$. The assumption that $\alpha _i$ are distinct guarantees $\det ({\mathbb {M}})=\prod _{1\le k< l\le M}(\alpha _l-\alpha _k)\ne 0$, equivalently, the invertibility of ${\mathbb {M}}$. Therefore, as a map from ${\mathbb {R}}^M$ to ${\mathbb {R}}^M$ assigning a probability distribution to a moment vector, ${\mathbb {M}}$ is bijective, i.e., different probability distributions must associate with different moment vectors, which also verifies the aforementioned Hausdorff moment problem that ${\textbf{p}}_i$ is uniquely determined by ${\textbf{m}}_i$ and vice versa from the perspective of linear algebra. Together with the fact that ${\textbf{p}}_i$ is the normalization of the predictor vector ${\textbf{x}}_i$ containing the medical records of the $i^{\textrm{th}}$ patient, this observation firmly declares the candidacy of the moment vector ${\textbf{m}}_i$ as the feature for medical decision making tasks.

Moreover, because the normalization procedure illustrated in (2) endows each ${\textbf{p}}_i$ with the property $\sum _{j=1}^Mp_{ij}=1$, if $M-1$ components of ${\textbf{p}}_i$ are known to us, say the first $M-1$ components $p_{i1}$, $\dots$, $p_{i,M-1}$, then the remaining component can be explicitly calculated as $p_{iM}=1-\sum _{j=1}^{M-1}p_{ij}$, i.e., the freedom to determine an *M*-dimensional probability vector is of degree $M-1$. Consequently, the normalized clinical dataset $\{{\textbf{p}}_1,\dots ,{\textbf{p}}_N\}$ lies on a proper subspace of ${\mathbb {R}}^M$ of dimension at most $M-1$. Then, in practice, the use of moments up to some order $M'<M-1$ may be sufficient to make a strategic medical decision. In this case, the *moment kernel* as defined in (4) becomes an *M*-by-$M'$ matrix, which transforms high-dimensional predictor vectors to low-dimensional moment vectors while retaining all the information required for making a medical decision at a lower computational cost.

In addition, the moment kernel in (4) is independent of the data, and hence ${\textbf{A}}$ is a dummy random variable so that the sample space $\Omega$, containing the *M* outcomes of ${\textbf{A}}$, is completely free to choose. To inform strategic medical decisions, we seek a construction of $\Omega$ suitable for ranking the relative contribution of feature vectors, which we discuss in the next section.

### HSIC lasso

Because each moment $m_{ik}$ in (3) is a weighted sum of the normalized predictors $p_{ij}$ with the weights $\alpha _j^k$, the choice of the sample space $\Omega =\{\alpha _1,\dots ,\alpha _M\}$ boils down to the determination of the weights. Naturally, predictors with larger weights acknowledge greater importance to the decision making process.

To this end, we formulate the task of searching for $\Omega$ as a feature importance ranking (FIR) problem^22^, to assign larger weights to more informative predictors. In particular, we will apply the Hilbert Schmidt Independence Criterion (HSIC) Lasso algorithm formulated as^23^

5 $$\begin{aligned} \begin{aligned} \min _{\alpha \in {\mathbb {R}}^M} \quad&\frac{1}{2} \Vert \bar{{\textbf{L}}} - \sum \limits _{j=1}^{M} \alpha _j \bar{{\textbf{K}}}^{(j)} \Vert _{\text {Frob}}^2 + \lambda \Vert \alpha \Vert _1, \\ \text {s.t.} \quad&\alpha _1, \ldots , \alpha _M \ge 0, \end{aligned} \end{aligned}$$

where $\Vert \cdot \Vert _{\text {Frob}}$ is the Frobenius norm of matrices, i.e., $\Vert {\textbf{A}}\Vert _{\text {Frob}}=\sqrt{\sum _{i=1}^m\sum _{j=1}^nA_{ij}^2}$ for any ${\textbf{A}}\in {\mathbb {R}}^{m\times n}$ with the (*i*, *j*)-entry $A_{ij}$, and $\Vert \alpha \Vert _1=\sum _{i=1}^M|\alpha _i|$ is the $\ell ^1$-norm of the vector $\alpha =(\alpha _1,\dots ,\alpha _M)$, and $\lambda >0$ is a constant controlling the sparsity of the solution. Moreover, $\bar{{\textbf{K}}}^{(j)} = \Gamma {\textbf{K}}^{(j)} \Gamma$ and $\bar{{\textbf{L}}} = \Gamma {\textbf{L}} \Gamma$ are centered Gram matrices with the entries ${\textbf{K}}_{m,n}^{(j)} = k(p_{j,m},p_{j,n})$ and ${\textbf{L}}_{m,n} = l(y_m,y_n)$ defined by using some kernel functions *k* and *l*, where $y_i$ denotes the class label of the $i^{\textrm{th}}$ patient and $\Gamma = {\textbf{I}}_N - \frac{1}{N} {\textbf{1}}_N {\textbf{1}}^{\top }_N$ is the centering matrix. Moreover, for memory and computational efficiency, we use Block HSIC Lasso^24^ in our experiments.

**Figure 1:**
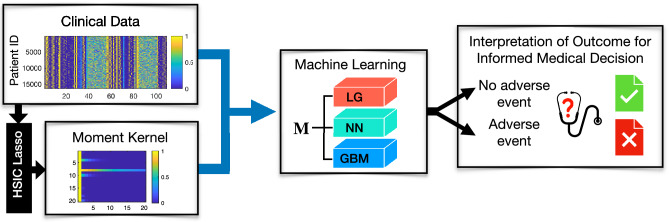
The pipeline for our feature extraction method through moments. HSIC Lasso is applied to the clinical data to obtain feature importance score (weights) for each feature. The weights are then used to form the moment kernel defined in (4). The efficient representation ${\textbf{M}}$ of the original clinical data is then generated through the moment kernel operation. ${\textbf{M}}$ will then be used in three machine learning algorithms: logistic regression (LR), neural networks (NNs), and gradient boosting machines (GBMs). The prediction of the machine learning algorithms further informs medical decision making.

The pipeline of our feature extraction framework through moments is summarized in Fig. 1. The following case studies further show that the performance of the classification using the moment vectors ${\textbf{m}}_i$, generated from the moment kernel in (4), as features is comparable to those using other features with reduced computation time and increased robustness.

### Case studies

#### Real-world data

**Figure 2:**
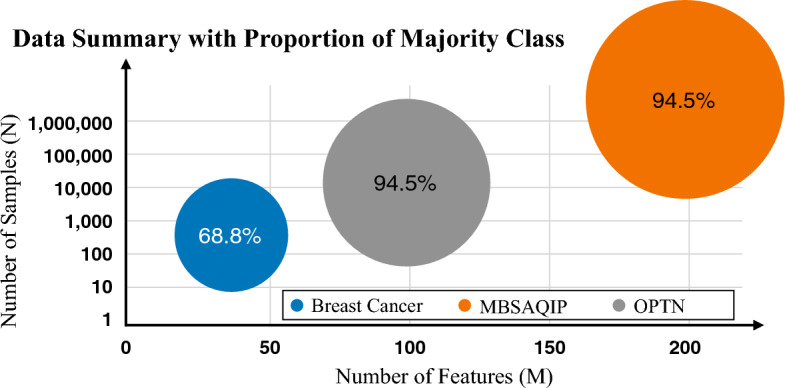
Summary description of the datasets.

We present three real-world datasets, including making informed decisions about breast cancer surgery, weight loss surgery, and liver transplant surgery by using pre-surgery patients’ clinical data. Specifically, we use the breast cancer dataset from the UCI Machine Learning Repository^25^, which is a publicly available database with open access, for classification of breast cancer recurrence events. The Metabolic Bariatric Surgery Accreditation and Quality Improvement Program (MBSAQIP) dataset^26^ is used for classification of the incidence of catastrophic events, including death, unplanned admission to ICU, and at least one re-operation within 30 days after weight loss surgery, and the Organ Procurement and Transplantation Network (OPTN) dataset^27^ is used for classification of graft failure following a liver transplant surgery. Both MBSAQIP and OPTN datasets are publicly available and accessible upon request. The three datasets are briefly summarized in Fig. 2, and a more detailed summary of each dataset is included in the Supplemental Material.

All procedures included in this work were performed in accordance with the relevant regulations and guidelines, and all informed consents were obtained before the admission to respective medical institute took place. The breast cancer dataset was collected from the University Medical Center, Institute of Oncology, Ljubljana, Yugoslavia. The MBSAQIP dataset is a Health Insurance Portability and Accountability Act (HIPAA)-compliant data file containing cases submitted to the MBSAQIP Data Registry, which contains patient-level, aggregate data and does not identify hospitals, health care providers, or patients. The OPTN dataset is collected via an online Web application. Transplant professionals from hospitals, histocompatibility (tissue typing) laboratories, and organ procurement organizations located across the United States use the application to manage their list of waiting transplant candidates, access and complete electronic data collection forms, add donor information and run donor-recipient matching lists, access various transplant data reports and policies. No organs/tissues were procured from prisoners.

For each dataset, we use an 80% training and 20% testing split, and explore three types of feature engineering schemes ${\textbf{M}}$, ${\textbf{X}}$ and ${\textbf{X}}(\alpha )$ for classification, where ${\textbf{M}}$ contains the features generated by the moment kernel in (4), ${\textbf{X}}$ presents the preprocessed data (obtained by normalization and one-hot encoding), and ${\textbf{X}}(\alpha )$ consists of the features in ${\textbf{X}}$ after feature selection. Each set of features are used to train three classifiers, including logistic regression (LR), artificial neural networks (NNs), and gradient-boosting machines (GBMs), and we examine the computation time and the area under the Receiving Operator Characteristic (AUC) curve for the testing data.

As shown in Fig. 2, all three datasets are imbalanced. Imbalanced datasets contain significantly uneven class labels. To address the issue of data imbalance, we adopt the following accommodations in the training phase. For LR, observation weights are added according to the ratio of class-imbalance; for NNs, an error weight based on class distribution is added to punish the misclassification of the minority label; for GBMs, we use RUSBoost^28^, a boosting method well-known for its robustness to class imbalance, to learn from the skewed training data. In the testing phase, the AUC is naturally immune to class imbalance so that the classification performance accurately reflects the performance of the classifiers.

#### Synthetic data

Finally, we also test robustness to number of samples, features, noise and missing values for each preprocessing scheme using five experiments : (a) noise-free data, (b) data with signal-to-noise ratio $SNR = 20$, (c)-(e) missing data in significant features, in which experimental data are synthetically generated. In each of these experiments, we have 10,000 samples ($N=10,000$) and 2,000 predictors ($M=2,000$), and among the 2,000 predictors only 5 of them are causal; namely, only five of them really contribute to the output labels, and the other 1,995 features are randomly generated, which are independent of the output labels. Causal features are generated by two Gaussian distributions with mean $(\mu _{i1},\mu _{i2}) = (0.3i, 0.7i), i = 1, \ldots , 5$ and the same variance $\sigma ^2 = 1$, representing data from two different classes.

The order of moments is set to be a tunable hyper-parameter, which, in this work, is chosen to optimize the AUC via cross-validation for each of the ML algorithms. Note that increasing the number of moments does not necessarily improve classification performance, as more moments (features) used may lead to a higher chance of overfitting. To illustrate this idea, in Fig. 5, we use another synthetic dataset with 100 observations and 100 predictors. The AUC results are given using ${\textbf{M}}$ with the order of moment *M* taken up to 20.

#### Computational resources

All the case studies were executed using MATLAB on a Windows 10 operating system with i5-7600K 3.80 GHz CPU and 16 GBM RAM memory.

## Results

### Real world data

**Figure 3:**
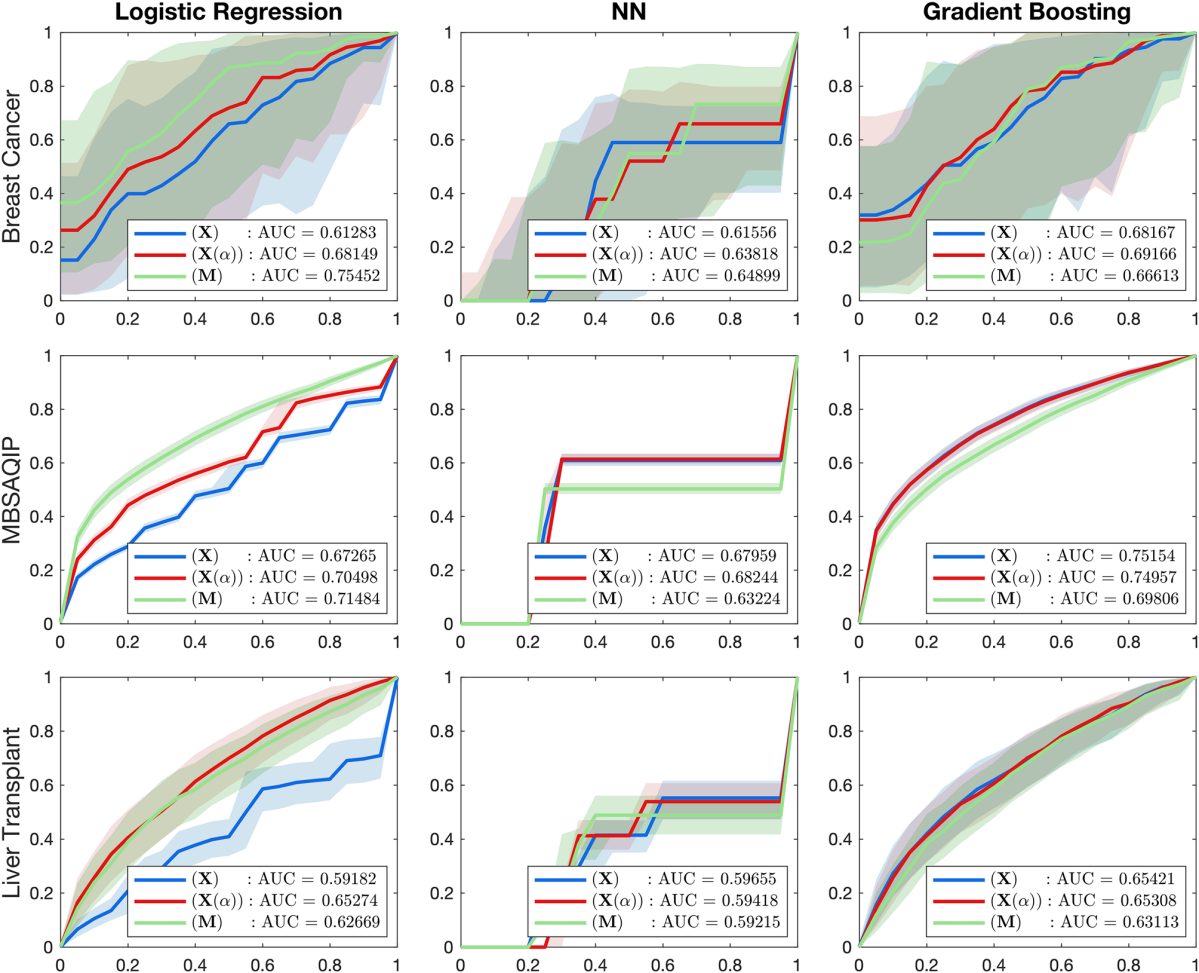
ROC curve for the classification task using (1) ${\textbf{X}}$: the original dataset, (2) ${\textbf{M}}$: the reduced dataset, and (3) ${\textbf{X}}(\alpha )$: the dataset that contains only features identified by HSIC Lasso. The proposed method is capable of selecting non-redundant features that offset the influence of noise.

The AUC results for three real world data are shown in Fig. 3, along with a summary of the results and comparison to other published works in Table 1. We determine the number of features required for ${\textbf{M}}$ by optimizing the AUC via cross-validation. For the breast cancer dataset, the percentage of training time saved using ${\textbf{M}}$ compared to ${\textbf{X}}$ is the highest for LR (64%), followed by NN (51%) and GBM (19%). Our preprocessing scheme ${\textbf{M}}$ is also more efficient than $\mathbf {X(\alpha )}$. We observe that across all 3 algorithms, ${\textbf{M}}$ generated AUC scores of 0.75, 0.65, and 0.70, for the LR, NN, and GBM models which were as high or higher than the next best performing method ${\textbf{X}}(\alpha )$. Using ${\textbf{M}}$ also required only 5 features, compared to 38 and 25 for ${\textbf{X}}$ and ${\textbf{X}}(\alpha )$. Compared to a published work^29^ that gives an highest accuracy of 72.7% using the C4.5 decision trees, which is based on the information gain of the raw features to split the decision trees, our method gives an improved accuracy of 75%..

For the MBSAQIP dataset, the time required to train using ${\textbf{M}}$ is substantially lower than both ${\textbf{X}}$ and ${\textbf{X}}(\alpha )$. The percentage of time saved for ${\textbf{M}}$ compared to ${\textbf{X}}$ is 99% for LR, 91% for NN, and 31.4% for GBM. We also observe comparable AUC testing performance using ${\textbf{M}}$. Using LR as the ML model, the AUC when using ${\textbf{M}}$ is second-highest, compared to 0.75 when using ${\textbf{X}}(\alpha )$. For NN and GBM, the AUC when using ${\textbf{M}}$ is slightly lower compared to ${\textbf{X}}(\alpha )$ (0.63 and 0.70 compared to 0.68 and 0.75), but the testing accuracy is nearly the same (75% and 74% compared to 82% and 84%). We also observe that the preprocessed data ${\textbf{M}}$ only used 30 features, compared to 163 and 131 for ${\textbf{X}}$ and ${\textbf{X}}(\alpha )$. In the previous work^30^, the authors integrated multiple machine learning models for the classification task. In essence, the ensemble of models is also a decision-tree-based algorithm, where enhanced features resulting from these machine learning algorithms are combined into an ensemble.

Lastly, for the liver transplant dataset, the percentage of time saved using ${\textbf{M}}$ is the most for LR (98%), followed by NN (59%) and then GBM (17%). Across all three ML algorithms, we observe similar AUC for each preprocessing scheme, where ${\textbf{M}}$ generates AUC scores of 0.63, 0.59, and 0.63 for LR, NN, and GBM, compared to the highest AUCs found with other preprocessing schemes of 0.66, 0.60, and 0.65 for the same models. Similarly to the other datasets, using ${\textbf{M}}$ as the preprocessing scheme required only 20 features compared to 99 and 91 features for ${\textbf{X}}$ and ${\textbf{X}}(\alpha )$. In a published work^31^ using a deep neural network on pre-surgical data, the authors included 202 features, even after feature selection.

To further compare the classification performance of the proposed MKM framework with other feature selection methods, in Table 2, we demonstrate the results of AUC performance obtained by applying several widely-used feature selection methods, including Chi-Square test^32^, minimum redundancy maximum relevance (MRMR)^33^, neighborhood component analysis (NCA)^34^, correlation-based feature selection (CFS), and BorutaShap^35^. In the table, *M* features with the highest importance weights are retained to construct ${\textbf{X}}(\alpha )$ using different feature selection methods. We observe that ${\textbf{M}}$ remains competitive among all other feature selection methods in terms of both classification performance and model parsimony.

In addition to ${\textbf{X}}(\alpha )$, we also compare the classification results obtained by using ${\textbf{M}}$ generated by the aforementioned feature selection methods. Similarly, we keep 5, 20, and 30 moments for the breast cancer, liver transplant, and MBSAQIP datasets, respectively, and perform 10-fold cross-validation for testing. The results are shown in Fig. 1 of the Supplemental Material, from which we observe that in most of the cases, different feature selection methods for generating ${\textbf{M}}$ result in similar performances, and the proposed HSIC has a slight advantage over the other feature selection methods. This illustrates the robustness of the feature extraction framework through the notion of moments. The extracted ${\textbf{M}}$ remains competitive in both model parsimony and classification performance regardless of the feature selection methods used.

**Table 1:** Classification AUC (accuracy) performance on testing data and model training time using the proposed feature selection methods and a comparison with the highest published in other works. The feature dimension of the dataset being trained (denoted as *M*) is also recorded in the table. ${\textbf{M}}$ represents the features generated by the moment kernel in (4); ${\textbf{X}}$ denotes the features generated by normalization and one-hot encoding; ${\textbf{X}}(\alpha )$ denotes important features obtained from HSIC Lasso (i.e., Eq. (5)).

Dataset	LR	Time(s) (% saved)	NN	Time(s) (% saved)	GBM	Time(s) (% saved)	$M^{*}$	Published works
Breast cancer								
${\textbf{X}}$	0.65 (63%)	0.027 ( - )	0.61 (60%)	0.126 ( - )	0.69 (70%)	0.575 ( - )	38	
${\textbf{X}}(\alpha )$	0.69 (67%)	0.041 ( - )	0.63 (67%)	0.083 (34%)	0.70 (71%)	0.500 (13%)	25	(72.7%)^29^
${\textbf{M}}$	0.75 (75%)	0.01 (64%)	0.65 (68%)	0.062 (51%)	0.70 (70%)	0.464 (19%)	5	
MBSAQIP								
${\textbf{X}}$	0.67 (83%)	101.79 ( - )	0.68 (74%)	124.4 ( - )	0.75 (85%)	8.418 ( - )	163	
$${\textbf{X}}(\alpha )$$	0.75 (75%)	78.1 (23%)	0.68 (74%)	124.0 (0.3%)	0.75 (84%)	7.942 (5.6%)	131	0.674^30^,$$^{a}$$
${\textbf{M}}$	0.71 (79%)	0.86 (99%)	0.63 (75%)	11.58 (91%)	0.70 (82%)	5.779 (31.4%)	30	
Liver Transplant								
${\textbf{X}}$	0.66 (68%)	2.340 ( - )	0.60 (64%)	0.657 ( - )	0.65 (75%)	0.981 ( - )	99	
$${\textbf{X}}(\alpha )$$	0.65 (66%)	0.466 (80%)	0.59 (64%)	0.783 ( - )	0.65 (75%)	0.980 (0.1%)	91	0.708^31^,$$^{b}$$
${\textbf{M}}$	0.63 (71%)	0.046 (98%)	0.59 (68%)	0.226 (59%)	0.63 (63%)	0.810 (17%)	20	

$^{a}$ The work has a different aim from ours: to predict the risk of 30-day readmission after bariatric surgery. While the published work focus on only one of the catastrophic events, we are also interested in the other 14 events that may occur post-surgery.

$^{b}$ The author apply deep neural networks (DNNs) to predict 90-day post- transplant mortality using preoperative variables and compared the performance to that of the Survival Outcomes Following Liver Transplantation (SOFT) and Balance of Risk (BAR) scores. The DNN exhibits optimal performance with 202 feature inputs, 5 hidden layers with 100 neurons each, resulting in a time complexity of roughly ${\mathcal {O}}(202^2 \cdot 100 \cdot 5 \cdot N)$, where *N* is the number of samples. Although the published DNN methods yield slightly superior results, our approach, which uses only 20 features, i.e., the order of moments, is more suitable for real-time applications due to its shorter training time. Specifically, the time complexity of our method amounts to ${\mathcal {O}}(20 \cdot N)$ for LR, ${\mathcal {O}}(20^2 \cdot 10 \cdot N)$ for NNs with 10 hidden layers, and ${\mathcal {O}}(20 t d \log N \cdot N)$ for GBMs. Here, *t* denotes the number of trees, and *d* represents the layer of the trees chosen. In our implementation, we chose $d = 10$ and $t = 50$, such that $20 t d \log N \approx 97,037 \ll 202 \cdot 100\cdot 5 = 20,402,000$.

**Table 2:** Comparison of classification AUC performance obtained by using other feature selection methods, including Chi-Square test, minimum redundancy maximum relevance (MRMR), neighborhood component analysis (NCA), correlation-based feature ranking, and the BorutaShap method, where *M* denotes the number of features used in the classifier and ${\textbf{M}}$ denotes features generated through the proposed MKM framework under the HSIC Lasso feature selection method.

Dataset ${\textbf{X}}(\alpha )$	Breast Cancer		Liver Transplant		MBSAQIP	
	AUC (95% CI)		AUC (95% CI)		AUC (95% CI)	
LR	$M=30$	$M=35$	$M=95$	$M=99$	$M=140$	$M=160$
HSIC Lasso	**0.67** (0.63, 0.71)	0.65 (0.63, 0.66)	**0.63** (0.59, 0.67)	0.62 (0.54, 0.71)	0.70 (0.69, 0.71)	0.70 (0.69, 0.71)
Chi-square	0.63 (0.61, 0.65)	0.64 (0.62, 0.66)	0.62 (0.55, 0.69)	0.59 (0.51, 0.68)	**0.71** (0.70, 0.72)	0.51 (0.50, 0.52)
MRMR	0.63 (0.59, 0.66)	0.61 (0.58, 0.62)	0.58 (0.50, 0.67)	0.60 (0.51, 0.70)	0.69 (0.67, 0.71)	0.69 (0.68, 0.70)
NCA	**0.67** (0.63, 0.71)	0.62 (0.60, 0.64)	0.55 (0.53, 0.57)	0.55 (0.53, 0.58)	0.51 (0.50, 0.52)	0.50 (0.50, 0.51)
Correlation	0.63 (0.61, 0.65)	0.61 (0.59, 0.64)	0.59 (0.52, 0.67)	0.64 (0.63, 0.66)	0.52 (0.51, 0.52)	0.51 (0.50, 0.52)
BorutaShap	0.66 (0.63, 0.69)	0.66 (0.62, 0.70)	0.59 (0.54, 0.65)	0.62 (0.60, 0.64)	0.52 (0.52, 0.53)	0.51 (0.50, 0.52)
**M** performance	$M= 5$, AUC $=0.75$		$M= 20$, AUC $= 0.63$		$M= 30$, AUC $= 0.71$	
NN						
HSIC Lasso	0.59 (0.53, 0.66)	0.62 (0.59, 0.64)	0.60 (0.58, 0.62)	0.60 (0.59, 0.61)	0.63 (0.62, 0.64)	0.60 (0.59, 0.62)
Chi-Square	0.63 (0.61, 0.65)	0.64 (0.62, 0.66)	**0.61** (0.59, 0.63)	0.60 (0.59, 0.62)	**0.64** (0.63, 0.65)	0.63 (0.61, 0.65)
MRMR	0.62 (0.59, 0.65)	0.63 (0.61, 0.66)	0.58 (0.50, 0.66)	0.60 (0.51, 0.70)	0.49 (0.48, 0.49)	0.52 (0.50, 0.54)
NCA	**0.67** (0.63, 0.71)	0.65 (0.62, 0.68)	**0.61** (0.60, 0.61)	0.59 (0.58, 0.61)	0.61 (0.59, 0.63)	0.61 (0.59, 0.62)
Correlation	0.64 (0.62, 0.66)	0.63 (0.61, 0.65)	0.60 (0.58, 0.62)	**0.61** (0.60, 0.62)	0.60 (0.59 0.61)	0.62 (0.60, 0.63)
BorutaShap	**0.67** (0.64, 0.70)	0.59 (0.56, 0.64)	0.60 (0.58, 0.62)	0.59 (0.59, 0.60)	0.60 (0.59, 0.61)	0.61 (0.60, 0.62)
**M** performance	$M= 5$, AUC $= 0.65$		$M= 20$, AUC $= 0.59$		$M= 30$, AUC $= 0.63$	
GBM						
HSIC Lasso	**0.69** (0.64, 0.74)	0.68 (0.63, 0.72)	0.64 (0.62, 0.66)	0.64 (0.63, 0.65)	0.69 (0.65, 0.72)	0.70 (0.67, 0.72)
Chi-Square	0.68 (0.66, 0.70)	0.68 (0.64, 0.71)	**0.65** (0.63, 0.67)	0.64 (0.62, 0.66)	0.70 (0.69, 0.71)	0.72 (0.69, 0.74)
MRMR	0.68 (0.63, 0.72)	0.68 (0.65, 0.71)	0.64 (0.62, 0.66)	0.64 (0.62, 0.66)	0.69 (0.68, 0.70)	0.71 (0.69, 0.72)
NCA	0.60 (0.55, 0.66)	0.58 (0.55, 0.62)	0.64 (0.62, 0.66)	**0.65** (0.63, 0.66)	0.70 (0.69, 0.71)	0.71 (0.69, 0.73)
Correlation	0.68 (0.65, 0.71)	0.67 (0.64, 0.71)	0.64 (0.62, 0.66)	**0.65** (0.63, 0.66)	0.70 (0.69, 0.72)	0.71 (0.70, 0.72)
BorutaShap	0.68 (0.63, 0.72)	0.66 (0.62, 0.70)	0.64 (0.62, 0.66)	0.64 (0.62, 0.66)	0.70 (0.69, 0.71)	**0.72** (0.69, 0.74)
**M** performance	$M= 5$, AUC $= 0.70$		$M= 20$, AUC $= 0.63$		$M= 30$, AUC $= 0.70$	

### Synthetic dataset

In all of the analyses using the synthetic dataset, we chose HSIC Lasso as our feature selection method to investigate the computation efficiency of the MKM framework for its robustness in performances across different real-world datasets. As summarized in Table 3, for our synthetic dataset with $N=10{,}000$ observations and $M=2,000$ predictors, in all of the scenarios studied, using ${\textbf{M}}$ consistently outperformed ${\textbf{X}}$ and ${\textbf{X}}(\alpha )$ in both the AUC score and the time required for training. The confidence intervals are also tighter for the ${\textbf{M}}$ cases.

**Figure 4:**
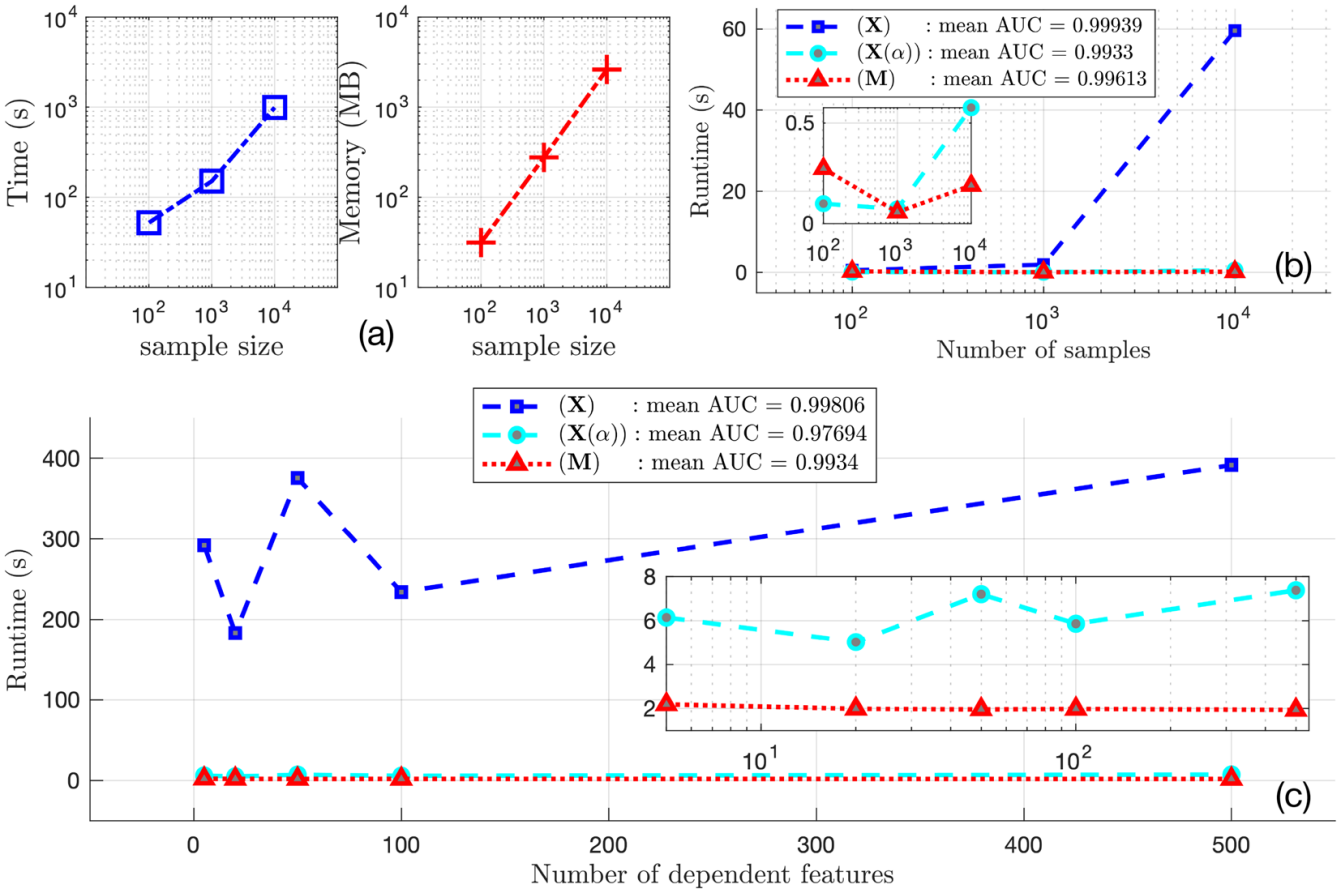
(**a**) Computational resources required by ${\textbf{X}}$, ${\textbf{X}}(\alpha )$ and ${\textbf{M}}$ for 10 bootstraps. (Left) Time elapsed during the training and test process. (Right) Memory used under a single-core scenario. (**b**) Runtime versus the number of samples using ${\textbf{X}}$, ${\textbf{X}}(\alpha )$ and ${\textbf{M}}$. The mean AUC score is calculated through averaging all of the bootstrapping cases. The zoomed-in figure shows only the case ${\textbf{M}}$ and ${\textbf{X}}(\alpha )$, from where we can still observe that runtime of ${\textbf{M}}$ does not increase with the sample size. (**c**) Runtime versus the number of features using ${\textbf{X}}$, ${\textbf{X}}(\alpha )$ and ${\textbf{M}}$. The mean AUC score is calculated through averaging all of the bootstrap cases. The zoomed-in figure shows ${\textbf{M}}$ and ${\textbf{X}}(\alpha )$. They are both immune to increasing features, but clearly ${\textbf{M}}$ has an edge over ${\textbf{X}}(\alpha )$ in runtime.

The synthetic dataset’s runtime performance is summarized in Fig. 4. Figure 4 panel (a) shows the memory usage (in MB) for each preprocessing scheme. Overall, using ${\textbf{M}}$ performs as well or better than ${\textbf{X}}$ and ${\textbf{X}}(\alpha )$ for both runtime and memory usage. Moreover, as shown in Fig. 4b, with the number of samples increasing from 100 to 10,000, using ${\textbf{M}}$ not only guarantees the shortest running time but also almost keeps the running time from increasing. The situation remains the same for the case of increasing the number of features as illustrated in Fig. 4c.

**Figure 5:**
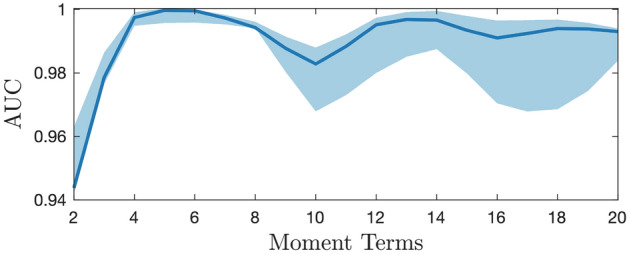
Number of moment terms v.s. AUC performance with 95% confidence interval for the synthetic dataset. In particular, 5-order moments give the optimal AUC performance of 0.9997, and increasing the order of moments order compromises the AUC performance due to overfitting.

The effect of moment order on the classification performance is summarized in Fig. 5. We observe that moments up to order 5 generate the best classification performance while adding orders higher than 5 gives sub-optimal performances. In this example, higher orders are redundant for approximating the original distribution of the datasets and result in overfitting.

**Table 3:** Classification AUC performance and model using ${\textbf{M}} \in {\mathbb {R}}^{N \times 5}$, ${\textbf{X}} \in {\mathbb {R}}^{N \times M}$ and ${\textbf{X}}(\alpha ) \in {\mathbb {R}}^{N \times k}$ under five different scenarios: (a) Noise-free data with perfect measurement, (b) data with signal-to-noise ratio $SNR = 20$, and (c)-(e) missing data in the five selected features. Mean AUCs and the respective confidence interval are also reported. The definitions of ${\textbf{M}}$, ${\textbf{X}}$, and ${\textbf{X}}(\alpha )$ are identical to those presented in Table 1.

	AUC	95% CI	Time(s)
(a) Noise-free data with perfect measurement			
${\textbf{M}}$	0.8666	(0.8311, 0.9000)	26.1505
${\textbf{X}}$	0.8209	(0.6880, 0.8810)	107.1619
${\textbf{X}}(\alpha )$	0.8209	(0.6880, 0.8810)	33.9519
(b) data with $SNR = 20$			
${\textbf{M}}$	0.8628	(0.8176, 0.9000)	31.0694
${\textbf{X}}$	0.8330	(0.7838, 0.8750)	97.7948
${\textbf{X}}(\alpha )$	0.8330	(0.7838, 0.8750)	25.5920
(c) missing data in one of the five selected features			
${\textbf{M}}$	0.8538	(0.8148, 0.8929)	33.5497
${\textbf{X}}$	0.8330	(0.7905, 0.8810)	105.3663
${\textbf{X}}(\alpha )$	0.8357	(0.7905, 0.8810)	32.8259
(d) missing data in two of the five selected features			
${\textbf{M}}$	0.8256	(0.7901, 0.8611)	37.4118
${\textbf{X}}$	0.8152	(0.7531, 0.8600)	101.4819
${\textbf{X}}(\alpha )$	0.8152	(0.7531, 0.8600)	32.0720
(e) missing data in three of the five selected features			
${\textbf{M}}$	0.7903	(0.7437, 0.8512)	36.5180
${\textbf{X}}$	0.7498	(0.5374, 0.8512)	105.2210
${\textbf{X}}(\alpha )$	0.7737	(0.7443, 0.8512)	37.5974

## Discussion

We present a feature extraction framework utilizing the notion of moments to construct a low-dimensional representation of the original high-dimensional dataset. The strength of this framework lies in its time and memory efficiency over the traditional HSIC Lasso method that is widely used for feature selection from large datasets. Through the representation of a moment kernel, in which the zero-weighting of the unimportant features and enlarging the useful ones are combined in one simple operation, we enhance the resistance of ML algorithms to noise and missing data, both of which are common problems encountered when we tackle real-world datasets. The training time for an ML model can be reduced by up to 99%, depending on the model used, while model performance is not compromised.

### Personalized medical decision making

It is common to treat each medical predictor as a random variable for leveraging its statistical properties, e.g., the probability distribution, analyzed over the sample space of the target population, e.g., patients diagnosed as suffering from some specific diseases, to assist medical decision making^36,37^. In our framework, the roles of patients and predictors are reversed: we formulate each patient as a probability distribution over the sample space consisting of the medical predictors. As a result, the analysis becomes patient-centered, and more importantly, our framework then gives rise to a patient-first and personalized medical decision making workflow.

The decisive factors affecting medical decisions in our model are the moment vectors output from the moment-kernel in (4). As defined in (3), moment vectors are obtained by taking expectations, i.e., averages, with respect to the probability distributions representing patients’ medical records. This indicates that decisions made by our machine are based on patients’ over health conditions by comprehensively evaluating all the medical predictors. Moreover, the integration of FIR in the feature extraction (moment vector computation) procedure further increases the influence of a small number of crucial predictors in making medical decisions. From the perspective of ML theory, this is effective to avoid overfitting so that our algorithms are expected to be more generalizable, which we explain in more detail in the following section.

### Model parsimony

Our results demonstrate that using the moment kernel requires significantly fewer features to achieve comparable or better performance than other feature extraction methods. This aptly demonstrates the effectiveness of the moments to represent the structures of the original datasets, the main reason for which is the averaging nature of the moments. Recall the definition in (3), each moment is a weighted sum of the normalized predictors of a patient, and hence depends on all the data collected for the patient, indicating its ability to reflect the overall structure of the data. On the other hand, as validated by the Hausdorff moment problem mentioned in the Methods section^21^, the collection of all the moments is also able to characterize the data in a comprehensive way, not only in an averaging way. As a result, a small number of the moments that document enough information for the classification task is already sufficient. In addition, the utilization of HSIC Lasso further outputs a sparse vector $\alpha$, which successfully reduces the dimension of the moment kernel. These characteristics make the developed moment kernel-based learning framework a parsimonious model with great computational efficiency and generalizability as discussed below.

### Performance robustness

Across all the case studies, the performance of the classifications using moment features is either comparable or outperforms those using other features, regardless of the choices of classifiers. In part, the robustness of our method across different datasets is the manifestation of its generalizability in ML terminology. In particular, deep NNs are known famous for the extraordinary power to derive conclusions from complex datasets even without any feature selection, that is, the preprocessing scheme ${\textbf{X}}$ in our notation. However, for all three datasets, the vanilla LR with ${\textbf{M}}$ can outperform NN. Together with the robustness to different classification algorithms, the developed moment method has the potential to serve as a universal ML-assisted medical decision making workflow.

Another significant advantage of our method is the robustness to noise and data loss. As illustrated in Table 3, with the degree of data loss increasing and the effect of noise coming in, although the performance drops, using ${\textbf{M}}$ consistently produces the best results compared to ${\textbf{X}}$ and ${\textbf{X}}(\alpha )$. These are also due to the averaging operation in the computation of moments, which smooths out the data, and hence is able to neutralize the effect of noise and compensate for missing values.

### Computational efficiency

Unarguably, the most significant advantage of utilizing moment features is the high computational efficiency. This is also mainly due to the parsimony of the moment-based model. In most of the cases in the case studies, by using moments as features, the model training time are dramatically reduced, e.g., for the MBSAQIP and liver transplant datasets, the time to train LR are reduced by 99% and 98%, respectively.

On the other hand, for the synthetic data, in addition to having a shorter running time comparable with ${\textbf{X}}(\alpha )$, ${\textbf{M}}$ also achieves the lowest memory consumption. Moreover, we also observe that the rates of increase in running time for ${\textbf{M}}$, as well as ${\textbf{X}}(\alpha )$, are remarkably small with the numbers of samples and features increasing, and ${\textbf{M}}$ persists to be the model with the shortest running time. In particular, for ${\textbf{M}}$ with respect to the number of dependent features, the increase of runtime is almost 0 even with the number of casual features having increased by a factor of 100. In summary, using moment features for learning tasks will not suffer from computational burden, which further claims the suitability of ${\textbf{M}}$ for tackling large-scale complex medical datasets.

### Limitations

Note that because each moment is a linear combination of the weights $\alpha _i$ output from the importance ranking algorithm HSIC Lasso, the importance of the predictors to the medical decisions may not be directly recognizable from the moments, which constitutes a possible limitation of the proposed moment kernel machine.

## Conclusion

We develop a moment kernel machine to extract features for predicting surgical outcomes using existing clinical data to inform decision making. The kernel is constructed through the notion of moments, which is capable of transforming complicated clinical data to compact and meaningful representations while retaining information crucial to medical decision making. In particular, the developed machine not only provides informative predictions for medical decision-making, but also is preferable to existing methods in terms of computational efficiency, model parsimony, and robustness to noise. Finally, this moment kernel machine has the potential to be personalized based on the specific requirements of patients and physicians, which is a significant development in ML-aided decision making methods in medicine.

## Supplementary Information

Supplementary Information.
