# Supplementary material for: A moment kernel machine for clinical data mining to inform medical decision making
Source: Sci Rep. 2023 Jun 28;13:10459. doi: 10.1038/s41598-023-36752-7 (PMC10307844; doi:10.1038/s41598-023-36752-7)
Supplement: Supplementary file 1 — Supplementary Information. [file 41598_2023_36752_MOESM1_ESM.pdf]

Supplementary Material

A Moment Kernel Machine for Clinical Data Mining to Inform Medical Decision Making

1 A Summary table for the breast cancer dataset

Table 1: Features and categorical values in the breast cancer dataset

Variable	
Age	
	10-19, 20-29, 30-39, 40-49, 50-59, 60-69, 70-79, 80-89, 90-99
Menopause	
	lt40, ge40, premeno
Tumor-size	
	0-4, 5-9, 10-14, 15-19, 20-24, 25-29, 30-34, 35-39, 40-44,45-49, 50-54, 55-59
Inv-nodes	
	0-2, 3-5, 6-8, 9-11, 12-14, 15-17, 18-20, 21-23, 24-26,27-29, 30-32, 33-35, 36-39
Node-caps	
	Yes, No
Deg-malig	
	1, 2, 3
Breast	
	left, right
Breast-quad	
	left-up, left-low, right-up, right-low, central
Irradiation	
	Yes, No
Class	
	no-recurrence-events, recurrence-events

2 A Summary table for the MBSAQIP dataset

Among all of the variables in Table 2, four of them are adapted from the original variables from the MBSAQIP dataset: number of postoperative ventilator occurrences, number of postoperative pneumonia occurrences, number of postoperative sepsis occurrences, and number of postoperative septic shock occurrences, as given in the footnote c of Table 2.

Table 2: Categorical variables and catastrophic event outcomes among 200,374 patients undergoing bariatric surgery in the MBSQIP dataset

Categorical variable	
SEX, n (%)	
Female	161,583 (80.6)
Male	38,791 (19.4)
Age greater than 80, n (%)	
No	200,284 (99.9)
Yes	90 (0.1)
Race, n (%)	
American Indian or Alaska Native	822 (0.4)
Asian	90 (0.5)
Black or African American	822 (17.6)
Native Hawaiian or Other Pacific Islander	90 (0.3)
Unknown/Not Reported	822 (8.4)
White	90 (72.8)
Hispanic Ethnicity, n (%)	
No	157,984 (78.8)
Unknown	18,160 (9.1)
Yes	24,230 (12.1)
GERD, n (%)	
No	136,622 (68.2)
Yes	63,752 (31.8)
Limited ambulation, n (%)	
No	197,496 (98.6)
Yes	2,878 (1.4)

Continued on next column

Categorical variable	(continued)
History of Myocardial Infarction, n (%)	
No	197,879 (98.8)
Yes	2,495 (1.2)
Previous PCI/PTCA, n (%)	
No	196,692 (98.2)
Yes	3,682 (1.8)
Previous Cardiac Surgery, n (%)	
No	198,341 (99.0)
Yes	2,033 (1.0)
Pre-Op Hypertension requiring medication, n (%)	
No	107,918 (53.9)
Yes	92,456 (46.1)
Number of Hypertensive Medications, n (%)	
0	108,151 (54.0)
1	42,121 (21.0)
2	31,302 (15.6)
3+	18,800 (9.4)
Pre-Op Hyperlipidemia, n (%)	
No	157,777 (77.7)
Yes	44,597 (22.3)
Pre-Op Vein Thrombosis Requiring Therapy, n (%)	
No	197,037 (98.3)
Yes	3,337 (1.7)
Pre-Op Venous Stasis, n (%)	
No	198,581 (99.1)
Yes	1,793 (0.9)
Pre-Op Requiring or on dialysis, n (%)	
No	199,787 (99.7)
Yes	587 (0.3)
Pre-Op Renal Insufficiency, n (%)	
No	199,160 (99.4)
Yes	1,214 (0.6)
Pre-Op Therapeutic Anticoagulation, n (%)	

Continued on next column

Categorical variable	(continued)
No	194,576 (97.1)
Yes	5,798 (2.9)
Previous Obesity surgery/foregut surgery, n (%)	
No	169,039 (84.4)
Yes	31,335 (15.6)
Pre-Op Diabetes Mellitus, n (%)	
Insulin	15,455 (7.7)
No	151,839 (75.8)
Non-Insulin	33,080 (16.5)
Current Smoker within 1 year, n (%)	
No	183,885 (91.8)
Yes	16,489 (8.2)
Pre-Op Functional Health Status, n (%)	
Independent	198,101 (98.9)
Partially Dependent	1,182 (0.6)
Totally Dependent	1,091 (0.5)
Pre-Op history of COPD, n (%)	
No	197,235 (98.4)
Yes	3,139 (1.6)
Pre-Op Oxygen Dependent, n (%)	
No	198,968 (99.3)
Yes	1,406 (0.7)
History of Pulmonary Embolism, n (%)	
No	197,911 (98.8)
Yes	2,463 (1.2)
Pre-Op Obstructive Sleep Apnea, n (%)	
No	129,409 (64.6)
Yes	70,965 (35.4)
Pre-Op Steroid/Immunosuppressant Use for Chronic Condition, n (%)	
No	196,705 (98.2)
Yes	36,69 (1.8)
Previous Organ Transplant, n (%)	
No	199,760 (99.7)
	Continued on next column

Categorical variable	(continued)
Yes	614 (0.3)
Pre-Op IVC Filter, *n* (%)	
No	199,293 (99.5)
Yes	1,081 (0.5)
First Assist Training Level, *n* (%)	
Attending - Other	10,353 (5.2)
Attending - Weight Loss Surgeon	28,890 (14.4)
Minimally Invasive Surgery Fellow	17,777 (8.8)
None (no assist or scrub tech/RN only)	30,959 (15.5)
PA/NP/RNFA	75,048 (37.5)
Resident (PGY 1-5+)	37,347 (18.6)
Emergency Case, *n* (%)	
No	198,178 (98.9)
Yes	2,196 (1.1)
Postoperative Coma > 24 Hours, *n* (%)	
No	200,369 (>99.9)
Yes	5 (<0.1)
Intraoperative or Postoperative Stroke/Cerebral Vascular Accident (CVA), *n* (%)	
No	200,344 (>99.9)
Yes	30 (<0.1)
Intra-Op or Post-Op Cardiac Arrest Requiring CPR, *n* (%)	
No	200,282 (>99.9)
Yes	92 (<0.1)
Intra-op or Post-op Myocardial Infarction, *n* (%)	
No	200,308 (>99.9)
Yes	66 (<0.1)
Postoperative Pulmonary Embolism, *n* (%)	
No	200,142 (99.9)
Yes	232 (0.1)
Transfusion Intra-Op/Post-Op (72h of surgery start time), *n* (%)	
No	198,868 (99.3)
Yes	1,506 (0.7)

Continued on next column

Categorical variable	(continued)
Unplanned Intubation, n (%)	
No	200,086 (99.9)
Yes	288 (0.1)
Unplanned Admission to ICU within 30 days, n (%)	
No	198,783 (99.2)
Yes	1,591 (0.8)
At least one reoperation within 30 days of op, n (%)	
No	197,336 (98.5)
Yes	3,038 (1.5)
At least one readmission within 30 days of op, n (%)	
No	192,481 (96.1)
Yes	7,893 (3.9)
Death during Operation (Intra-Op Death) or Post-Op Death within 30 Days of Procedure, n (%)	
No	200,172 (96.1)
Yes	202 (3.9)
Number of Postoperative Ventilator (PV) occurrences[1], n (%)	
No occurrence	200,173 (99.9)
One or Two occurrences	201 (0.1)
Number of Postoperative Pneumonia (PP) occurrences[1], n (%)	
No occurrence	199,882 (99.8)
One or Two occurrence	492 (0.2)
Number of Postoperative Sepsis (PS) Occurrences[1], n (%)	
No occurrence	200,029 (99.8)
One or Two occurrence	345 (0.2)
Number of Postoperative Septic Shock (PSS) Occurrences[1], n (%)	
No occurrence	200,159 (99.9)
One or Two occurrence	215 (0.1)
Recoded Race[2], n (%)	
Non-White	37,673 (18.8)
Unknown/Not Reported	16,797 (8.4)
White	145,904 (72.8)
Catastropic Event[3], n (%)	

Continued on next column

Categorical variable	(continued)
No	189,272 (94.5)
Yes	11,102 (5.5)
	Concluded

[a]No patient had more than 2 events.

[b]Non-White consists of American Indian or Alaska Native, Asian, Black or African American, and Native Hawaiian or Other Pacific Islander. Categories were combined because of small cell counts in catastrophic events for races other than white

[c]A catastrophic event is defined as one of the following: postoperative coma > 24 hours, CVA, intra-op or post-op cardiac arrest requiring CPR, intra-op or post-op myocardial infarction, postoperative pulmonary embolism, transfusion intra-op/post-op, unplanned intubation, unplanned admission to ICU within 30 days, at least one reoperation within 30 days of op, at least one readmission within 30 days of op, and death during operation or post-op death within 30 days of procedure.

3 A Summary table for the OPTN dataset for liver transplant

Table 3: Categorical variables and outcomes of the liver transplant procedure among 16,379 patients in the OPTN dataset

Categorical variable	
Center volume, n (%)	
Low volume	4082 (24.9)
High volume	12297 (75.1)
Medical condition, n (%)	
Home	8,598 (52.5)
Inpatient	44,94 (27.4)
ICU	3,289 (20.1)
Unknown	1 ($\sim$0)
Gender, n (%)	
Male	99,43 (39.3)
Female	64,36 (60.7)
Blood type, n (%)	
A	6,082 (37.1)
B	2,164 (13.2)
AB	833 (5.1)
O	7,300 (44.6)
Drug treatment, n (%)	
No	136,622 (91.5)
Yes	63,752 (8.5)
Recipient life support pre- or at-transplant, n (%)	
No	14,453 (88.2)
Yes	1,926 (11.8)
Race-recoded, n (%)	
White	11,947 (72.9)
Black	1,610 (9.8)
Hispanics	2,113 (12.9)
Other	709 (4.4)

Continued on next column

Categorical variable	(continued)
Diabetes, n (%)	
No/Unknown	11,703 (71.4)
Yes	4,676 (28.6)
Human leukocyte antigen(HLA) mismatch level, n (%)	
Missing	8,536 (52.1)
Mismatch	7831 (47.9)
zMismatch	12 (∼0)
Etiology of liver disease, n (%)	
Fulminant/Biliary atresia/Malignant neoplasm	2,398 (14.6)
Metabolic disease/Cholestatic cirrhosis	1,874 (11.5)
Noncholestatic/other	12,107(73.9)
Deceased donor-antibody to HEP-C virus result, n (%)	
Positive	371 (2.3)
Negative	16,008 (97.7)
Education, n (%)	
None	62 (<1)
Some school (< 5y)	6 (<1)
Grade school (0–8y)	706 (4.4)
High school (9–12y)/GED	6,352 (38.8)
Bachelor degree	2,136 (13.1)
College/technical school	3,103 (19.0)
Graduate degree	892 (5.6)
Unknown	3,122 (19.1)
Insurance, n (%)	
Medicaid	1,143 (7)
Medicare	8,224 (50.1)
Other	6814 (41.7)
Private Insurance	198 (1.2)
Urban, n (%)	
No/Unknown	1,406 (8.6)
Yes	14,973 (91.4)
Was candidate listed for simultaneous kidney? n (%)	
No	13,258 (80.9)

Continued on next column

Categorical variable	(continued)
Yes	3,121 (19.1)
Ever had dialysis, n (%)	
No	11,237 (68.6)
Yes	5,142 (31.4)
Duration of the dialysis prior to transplant or censoring, n (%)	
None	12,085 (73.8)
≤ 60	814 (4.9)
≥ 60	1,143 (7)
Unknown	2,337 (14.3)
Income, n (%)	
1st quartile	4,028 (24.6)
2nd quartile	4,199 (25.6)
3rd quartile	3,993 (24.4)
4th quartile	3,655 (22.3)
Unknown	504 (3.1)
Surgery outcome, n (%)	
Success	15,445 (94.3)
Failure	934 (5.7)
	Concluded

4 Comparison of classification performance M among different feature selection methods

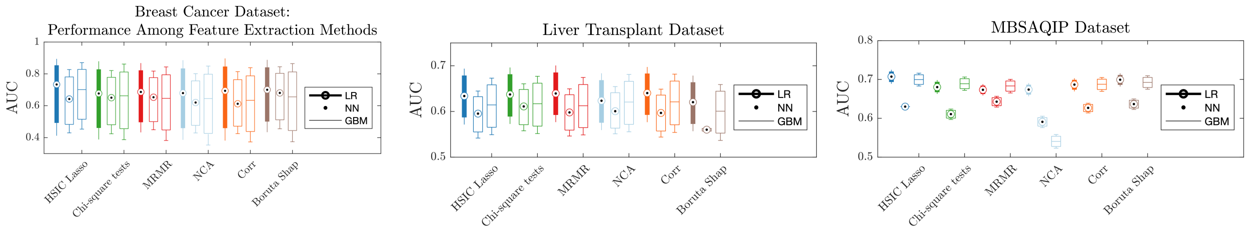

Figure 1: AUC performance obtained by extracting **M** under different feature selection methods. The same color groups represent classification results using LR, NN, and GBM methods (from left to right), and different colors represent different feature selection methods indicated in the horizontal axes. The bar region shows the 25th and 75th percentiles, respectively, and the central mark indicates the median.
